# Isocaloric switch from low-fat to Western high-fat diet triggers transcriptomic reprogramming and ectopic olfactory receptor responses

**DOI:** 10.1126/sciadv.aef5698

**Published:** 2026-08-19

**Authors:** Jonathan Bertram, Michail Lazaratos, Michael Kruse, Silke Hornemann, Alexander S. Mosig, Turid Frahnow, Martin Osterhoff, Andreas Busjahn, Anne-Cathrin Ost, Annette Schürmann, Olga Pivovarova-Ramich, Andreas FH Pfeiffer

**Affiliations:** ^1^ Charité – Universitätsmedizin Berlin, Corporate Member of Freie Universität Berlin and Humboldt-Universität zu Berlin, Department of Endocrinology and Metabolism, Berlin, Germany.; ^2^ German Center for Diabetes Research (DZD e.V.), München-Neuherberg, Germany.; ^3^ Department of Experimental Diabetology, German Institute of Human Nutrition Potsdam-Rehbruecke, Nuthetal, Germany.; ^4^ Institute of Biochemistry II, Jena University Hospital, Jena, Germany.; ^5^ Department of Molecular Metabolism and Precision Nutrition, German Institute of Human Nutrition Potsdam-Rehbruecke, Nuthetal, Germany.; ^6^ HealthTwiSt GmbH, Berlin, Germany.; ^7^ Institute of Midwifery Science and Interprofessional Perinatal Medicine, Leipzig University, Germany.; ^8^ Institute of Nutritional Sciences, University of Potsdam, Nuthetal, Germany.

## Abstract

Although Western diet (WD) rich in saturated fat is regarded as a trigger of metabolic disease and inflammation, the role of this diet in the absence of obesity is unclear. We studied the metabolic, inflammatory, anthropometric, and adipose tissue transcriptomic responses of 92 twins to 6 weeks of WD following 6 weeks of a healthy low-fat diet while maintaining stable body weight. The WD increased total, LDL and HDL cholesterol but not triglycerides or free fatty acids and induced transient and moderate increases of insulin resistance and some inflammatory markers after 1 week, which disappeared after 6 weeks. Extensive transcriptomic changes reflected restructuring of subcutaneous abdominal adipose tissue. Olfactory receptor (OR) mRNAs were increased in correlation with insulin resistance, liver, and visceral fat, cytokines like IL-18 and VEGF, but inversely related to adiponectin and extracellular NAMPT. In conclusion, WD triggers metabolic and transcriptomic reprogramming closely linked to ectopic OR responses.

## INTRODUCTION

Dietary recommendations from randomized controlled studies are based on biomarker responses to food components or dietary patterns linking them to favorable or unfavorable metabolic outcomes that are known to correlate with the risks of noncommunicable diseases. These biomarkers include dyslipidemia, dysglycemia, hypertension, abdominal fat accumulation, and obesity, which are summarized as metabolic syndrome. Subclinical chronic inflammation appears to represent an important further risk factor that increases risks for the development of cardiovascular disease, type 2 diabetes, and diverse cancers (*1*).

Western diet (WD) represents the prototype of an unhealthy diet due to its causal association with the development of lipid and glucose disturbances, hypertension, subclinical inflammation, and increased mortality (*2*–*4*). The molecular mechanisms induced by WD are mostly attributed to inflammatory responses. Mouse and human studies have shown that the inborn immune system is activated by circulating fatty acids and oxidized cholesterol via activation of the Toll-like receptors TLR2 and TLR4 (*2*, *5*, *6*). The subsequent signaling cascade activates the inflammasome, which drives the release of the interleukins IL-1β and IL-18. These cytokines induce a broad immune response and trigger chronic inflammation that is further enhanced by repeated stimulation, a condition termed trained immunity (*5*, *7*). Obesity and metabolic syndrome are known to enhance these responses (*8*).

However, high-saturated-fat diet intake in healthy individuals without excessive weight gain did not elicit inflammatory responses or insulin resistance in recent short-term intervention studies (*9*–*11*). Nonetheless, obesity is a frequent consequence of the WD and may be triggered by early responses to high-fat intake. The association of insulin resistance and inflammation with obesity thus appears to involve the combination of obesity and WD. To understand the role of nutrition, the effects of obesity and weight gain must be separated from the nutritional responses to WD.

In this study, representing a secondary analysis of the previously published NutriGenomic Analysis in Twins (NUGAT) trial (*12*–*14*), we aimed to identify transcriptomic, metabolic, and inflammatory responses upon intake of WD rich in saturated fat in healthy individuals in the absence of weight gain. We hypothesized that this may allow the identification of genetic pathways involved in the induction of the metabolic syndrome by WD. Since dietary habits vary widely, we started the study with 6 weeks of a healthy, low-fat diet (30% energy, %E), moderate protein (15%E) and carbohydrate (55%E) intake before the first clinical investigation day (CID1). This was intended to standardize the nutritional starting point. The participants then switched to a WD containing 45%E fat and 40%E carbohydrate and were examined after 1 week (CID2) and after 6 weeks (CID3) of this WD. The three time points were chosen to capture time courses of short and potentially transient responses as well as continuous alterations. Great care was taken to keep weight constant to avoid confounding by weight changes. The results show extensive transcriptomic responses in subcutaneous adipose tissue to WD. Metabolic and plasma inflammatory responses were monitored in parallel. Notably, pronounced changes were observed in adipose tissue gene expression. Unexpectedly, olfactory receptor (OR) genes ectopically expressed in adipose tissue were highly correlated with the metabolic and adipokine responses. These results suggest that ORs are closely linked to the negative properties of WDs.

## RESULTS

### Clinical measurements

The anthropometric and clinical characteristics of the participants are presented in Table 1 and fig. S1. The participants were mostly young, of normal weight, Caucasian origin, and healthy and details of the dietary intervention have been reported previously (*12*–*15*). Although there was a statistically significant increase in weight and body mass index (BMI), these changes were small (0.25 kg or 0.39% relative change from CID1 to CID3) (fig. S2). As expected, the WD caused a rapid increase in total, high-density lipoprotein (HDL) and low-density lipoprotein (LDL) cholesterol already after 1 week, which further increased after 6 weeks, thereby confirming the compliance of the participants (*16*). Free fatty acids decreased in response to the high-fat diet in this lean and healthy population, while triglycerides did not change in agreement with previous studies in healthy lean subjects (*11*, *16*, *17*).

**Table 1.** Clinical measurements across study time points. Values are presented as median [first quartile, third quartile] for each time point. Overall *P* values were assessed through a type III ANOVA with a χ2 test and *P* values for pairwise comparisons were obtained by comparing the estimated marginal means from the linear model and adjusted for multiple testing using the Benjamini-Hochberg method (*52*). The *P* values for pairwise comparisons are robust regarding heteroscedasticity and significant values are highlighted in bold.

Parameter	Time point			*P* value			
	CID1	CID2	CID3	CID 1v2	CID 2v3	CID 1v3	Overall
Weight [kg]	63.95 [58.60, 74.85]	63.40 [59.15, 74.45]	64.20 [58.70, 76.15]	0.265	**<0.001**	**<0.001**	**<0.001**
BMI [kg/m^2^]	22.19 [20.60, 24.27]	22.02 [20.60, 24.08]	22.46 [20.63, 24.43]	0.326	**<0.001**	**<0.001**	**<0.001**
WHR []	0.80 [0.75, 0.85]	0.80 [0.75, 0.84]	0.79 [0.74, 0.84]	0.606	0.499	0.377	0.269
IHL [%]	0.94 [0.56, 1.59]	0.84 [0.56, 1.77]	0.98 [0.54, 2.14]	0.815	0.815	0.815	0.679
HOMA-IR []	0.91 [0.66, 1.34]	1.07 [0.74, 1.45]	1.06 [0.63, 1.32]	**0.038**	0.281	0.396	0.081
Insulin [mU/l]	3.75 [2.73, 5.00]	4.91 [3.68, 6.26]	4.71 [3.00, 5.96]	**<0.001**	0.069	0.069	**0.001**
Glucose [mg/dl]	90.46 [84.51, 98.03]	93.25 [86.14, 99.65]	92.44 [85.60, 100.46]	0.719	0.385	0.385	0.315
AIRg [μU/ml]	212.37 [142.60, 313.20]	244.37 [175.59, 345.73]	255.32 [151.82, 356.30]	0.068	0.357	**0.039**	**0.026**
DI []	2,001.30 [1,464.60, 2,938.40]	2,377.00 [1,791.55, 3,150.35]	2,318.10 [1,675.20, 3,873.00]	0.372	0.431	0.315	0.188
CRP [mg/dl]	0.22 [0.05, 0.87]	0.22 [0.03, 0.81]	0.34 [0.08, 1.00]	0.695	0.066	0.066	**0.028**
Total cholesterol [mmol/l]	4.26 [3.64, 4.86]	4.48 [3.89, 4.95]	4.66 [3.95, 5.32]	**<0.001**	**<0.001**	**<0.001**	**<0.001**
HDL [mmol/l]	1.18 [1.02, 1.48]	1.27 [1.12, 1.56]	1.36 [1.12, 1.64]	**<0.001**	**<0.001**	**<0.001**	**<0.001**
LDL [mmol/l]	2.45 [2.09, 3.06]	2.70 [2.16, 3.25]	2.78 [2.25, 3.32]	**0.004**	**0.002**	**<0.001**	**<0.001**
TAG [mmol/l]	0.86 [0.64, 1.19]	0.81 [0.67, 1.05]	0.82 [0.61, 1.10]	0.332	0.799	0.42	0.285
NEFA [mmol/l]	0.59 [0.44, 0.77]	0.55 [0.46, 0.68]	0.49 [0.37, 0.62]	0.295	**0.006**	**<0.001**	**<0.001**
TNFα [pg/ml]	1.21 [1.01, 1.43]	1.36 [1.14, 1.66]	1.25 [1.06, 1.52]	**<0.001**	**0.016**	0.423	**0.001**
IL-6 [pg/ml]	0.85 [0.67, 1.34]	1.06 [0.75, 1.49]	0.86 [0.68, 1.16]	**0.043**	**0.002**	0.125	**0.002**
IL-8 [pg/ml]	5.07 [4.33, 6.34]	4.39 [3.64, 5.63]	4.98 [3.93, 6.41]	**0.001**	**0.007**	0.39	**0.002**
IL-18 [pg/ml]	207.90 [166.40, 271.10]	228.40 [180.15, 295.95]	207.95 [162.85, 297.80]	**<0.001**	0.309	0.165	**0.012**
IL-1ra [pg/ml]	188.60 [154.50, 248.40]	151.20 [125.60, 180.70]	183.40 [151.20, 223.80]	**<0.001**	**<0.001**	0.086	**<0.001**
IL-10 [pg/ml]	7.71 [5.86, 11.12]	6.11 [4.45, 7.94]	7.39 [4.96, 11.43]	**<0.001**	**<0.001**	0.481	**0.001**
MCP-1 [pg/ml]	322.55 [244.80, 377.80]	306.65 [248.60, 372.05]	300.55 [213.25, 351.35]	0.7	**0.01**	**0.021**	**0.014**
CCL3 [pg/ml]	17.90 [12.55, 22.30]	16.76 [12.80, 22.17]	16.47 [11.18, 22.74]	0.769	0.769	0.986	0.708
CCL5 [ng/ml]	31.00 [23.30, 42.31]	33.05 [25.90, 43.35]	29.00 [20.05, 38.15]	**0.03**	**<0.001**	0.059	**<0.001**
Adiponectin [μg/ml]	9.36 [7.59, 11.51]	9.40 [7.41, 11.59]	8.97 [6.81, 11.51]	0.148	0.148	0.53	0.149
eNAMPT [ng/ml]	0.55 [0.20, 1.05]	0.52 [0.25, 0.86]	0.49 [0.29, 1.01]	0.991	0.478	0.478	0.451
Chemerin [ng/ml]	132.70 [117.30, 149.50]	132.60 [119.70, 152.00]	139.40 [120.30, 154.70]	0.64	0.069	0.069	**0.026**
Progranulin [ng/ml]	53.56 [46.13, 59.94]	55.61 [50.75, 61.91]	50.02 [42.81, 55.59]	**<0.001**	**<0.001**	**0.001**	**<0.001**
FGF21 [pg/ml]	113.30 [71.00, 193.30]	120.15 [74.43, 217.60]	116.10 [73.66, 170.90]	**0.047**	**0.047**	0.989	**0.031**
PAI1 [U/ml]	6.75 [4.11, 11.38]	7.58 [4.76, 14.87]	7.94 [4.12, 15.50]	**0.006**	0.894	**0.01**	**0.004**
VEGF [pg/ml]	225.10 [137.55, 349.85]	246.80 [129.28, 346.76]	241.90 [141.60, 416.40]	0.498	**0.007**	**0.011**	**0.002**

The WD induced a small but significant increase in fasting plasma insulin after 1 week at CID2, which persisted at CID3, while fasting glucose remained unchanged. This resulted in a significant increase in insulin resistance, as measured by HOMA-IR at CID2. Insulin sensitivity and secretion determined by intravenous glucose tolerance tests (ivGTTs) regarding acute insulin response (AIRg) increased significantly at CID3, while the disposition index (DI), which is defined as the product of AIRg and the insulin sensitivity index, did not change significantly from CID1 to CID3 (Table 1) (*13*, *18*).

The levels of C-reactive protein (CRP) were low at baseline in the participants and increased slightly at CID3 (Table 1). The changes of the circulating inflammatory biomarkers and adipokines are grouped into cytokines, chemokines, and adipokines for better oversight and are listed in Table 1.

### Cytokines

The inflammasome-processed cytokine IL-1β was below detection limit of 0.023 pg/ml in ≥78.0% of the twins while IL-18 was measurable but only changed transiently in CID2. Similarly, the classical cytokines IL-6, tumor necrosis factor α (TNFα) and progranulin increased transiently after 1 week (CID2) and returned to or below baseline after 6 weeks of WD at CID3. Chemerin did not change. Vascular endothelial growth factor (VEGF) increased as we had previously reported (*12*).

### Anti-inflammatory cytokines

IL-10 and IL1ra, by contrast, decreased transiently at CID2 and returned to baseline levels at CID3.

### Chemokines

C-C motif chemokine ligand 2 (CCL2), also known as monocyte chemoattractant protein-1 (MCP1), decreased at CID3 while CCL5 (RANTES) increased transiently at CID2. CCL3, also known as macrophage inflammatory protein-1α (MIP1α), showed no change while IL-8 decreased transiently at CID2 and returned to baseline at CID3.

### Adipokines

Adiponectin and extracellular nicotinamide phosphoribosyltransferase (eNAMPT) showed no significant changes. Last, the hepatokine fibroblast growth factor 21 (FGF21) increased transiently at CID2, while plasminogen activator inhibitor-1 (PAI1) showed a significant increase at CID2, which continued to a lesser extent at CID3 (Table 1).

Adipose tissue is a major source of inflammatory mediators produced by immune and fat cells. We therefore estimated the immune cell infiltration of subcutaneous periumbilical adipose tissue from microarray gene expression profiles through the CIBERSORT analysis, which is well-established based on immune cell type–specific transcripts (*19*). Notably, the analysis did not indicate an increase in macrophages. There were moderate changes in CD4-T-cells and a slight increase in natural killer (NK) cells, indicating a mixed response of inborn and adaptive immune cells (table S1).

### Transcriptomic responses

**Fig. 1.**
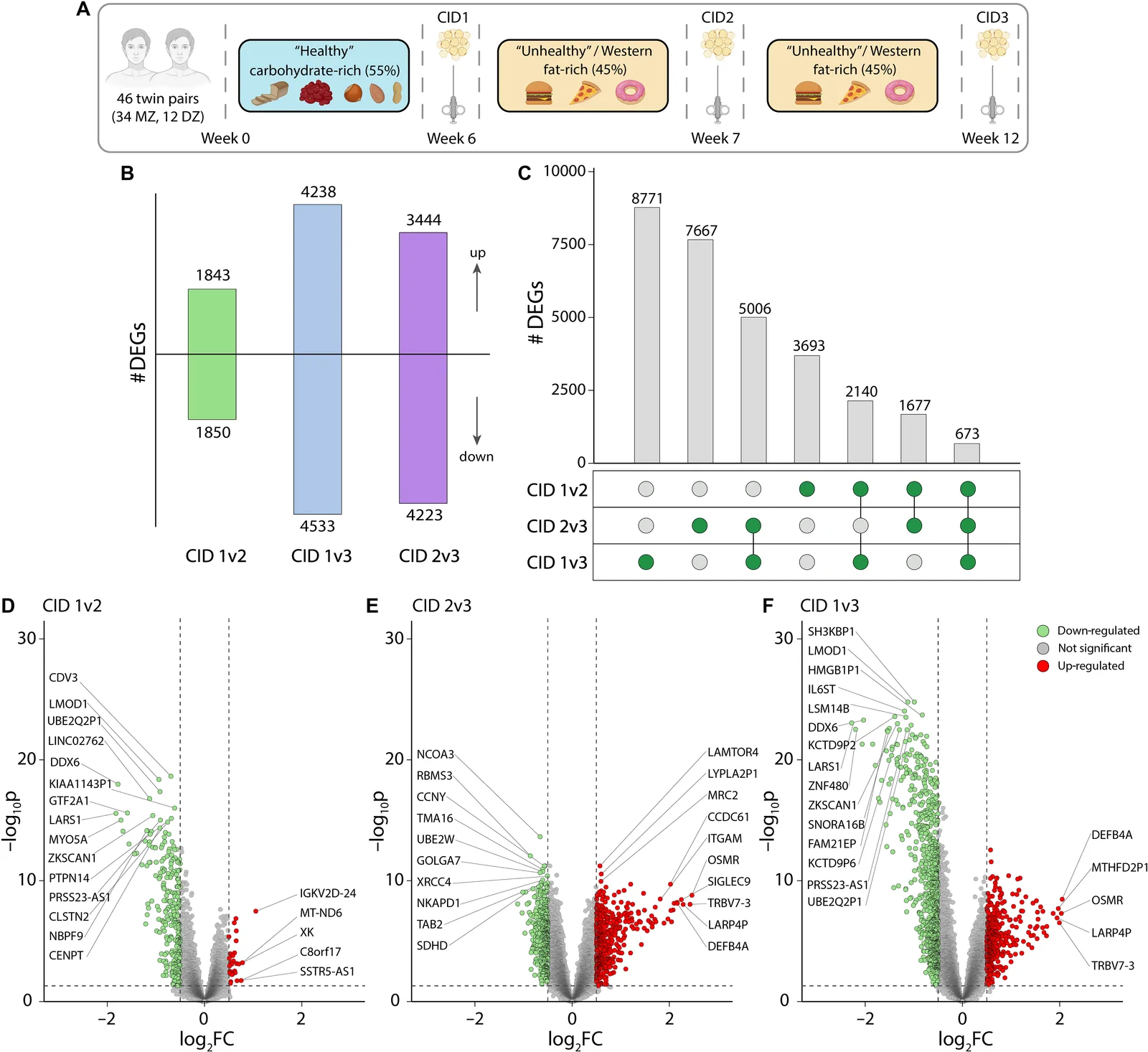
Transcriptomic analysis of subcutaneous periumbilical adipose tissue across CID1–3. (**A**) Graphical representation of the study design. The participants were subjected to a “healthy” low-fat diet for 6 weeks, after which the first adipose tissue biopsies were taken. A week of an “unhealthy”/Western fat-rich diet (45%) followed for 1 week, as well as an additional biopsy. Last, the fat-rich diet was sustained for an additional 5 weeks and the third biopsy was taken. (**B**) Bar chart of significant DEGs for each comparison between CIDs in either direction of regulation. (**C**) Upset plot showing the overlap of significant DEGs between comparisons, where the connected green dots indicate which overlaps are displayed. Bars represent the total number of significant DEGs regardless of direction of regulation for either a single comparison or an overlap of two or three comparisons. (**D** to **F**) Volcano plots for significant DEGs. Genes below the |log_2_FC| cutoff of 0.5 and adjusted *P* value of 0.05 are colored in grey, down-regulated genes above the cutoff are green, while up-regulated genes are red. Top genes with the most significant adjusted *P* values and/or highest FCs are labeled explicitly for each direction of regulation.

We next assessed responses of gene expression in subcutaneous periumbilical adipose tissue to the WD. The transcriptomic analysis comprised about 22,000 genes. Consumption of WD resulted in 3693 differentially expressed genes (DEGs) at CID2 as compared to CID1. Quantitatively similar DEGs were up- or down-regulated. Further extensive changes were seen between CID2 and CID3. Following a prolonged 5-week exposition to WD, 7667 DEGs were identified, with more genes being down-regulated than up-regulated. Last, after a total of 6 weeks of WD at CID3, 8771 DEGs were observed compared to CID1, again with approximately similar numbers of up- and down-regulated genes (Fig. 1, B and C).

Volcano plots (Fig. 1, D to F) show the top DEGs by fold change (FC) and *P* value. Cutoffs were set at a log_2_FC of 0.5 and an adjusted *P* value of 0.05. In CID1 versus CID2, most of the genes above the cutoff were down-regulated, with greater absolute FCs and more significant *P* values, many of which were associated with transcription and cell organization. For CID2 versus CID3, most genes were up-regulated and showed a greater absolute FC. Several top genes were related to inflammation and phagocytosis, although inflammation levels in most blood serum cytokines only changed transiently. CID1 versus CID3 showed a combination of both: The up- and down-regulated genes displayed meaningful FCs and were associated with regulation of transcription, cellular organization and inflammation (table S4).

Pathway enrichment analysis of DEGs further confirmed these associations. After the first week of WD in CID1 versus CID2, the top DEGs with the strongest changes were associated with regulation of transcription and cell organization (tables S5 and S6). Inspection of changes during the following 5 weeks of WD in CID2 versus CID3 showed a vast range of altered functions. The most significantly changed genes play a role in signaling cascades, sensory perception, and endocrine mechanisms but also in inflammatory processes (tables S7 and S8). The overall changes during the entire WD period in CID1 versus CID3, naturally, showed an even larger number of changes, including general regulation of cellular processes, chromatin organization, regulation of transcription, signaling cascades, and inflammation (tables S9 and S10).

To follow up on the discrimination between changes in up- and down-regulated genes, the DEGs were separated by direction of regulation, and the top 300 genes were again investigated with pathway enrichment analysis. The top down-regulated genes were in line with our previous findings, most significantly changing regarding cell processes, transcription, and inflammation. The top up-regulated genes in CID1 versus CID2 were highly associated with sensory perception, including perception of odorants and olfactory transduction but were also involved in the insulin resistance pathway. In CID2 versus CID3, the top up-regulated genes were mainly involved in inflammation, cell signaling and endocrine functions and CID1 versus CID3, expectedly, showed processes that also appear in CID1 versus CID2 and CID2 versus CID3 (tables S11 to S16).

### Weighted gene coexpression network analysis

**Fig. 2.**
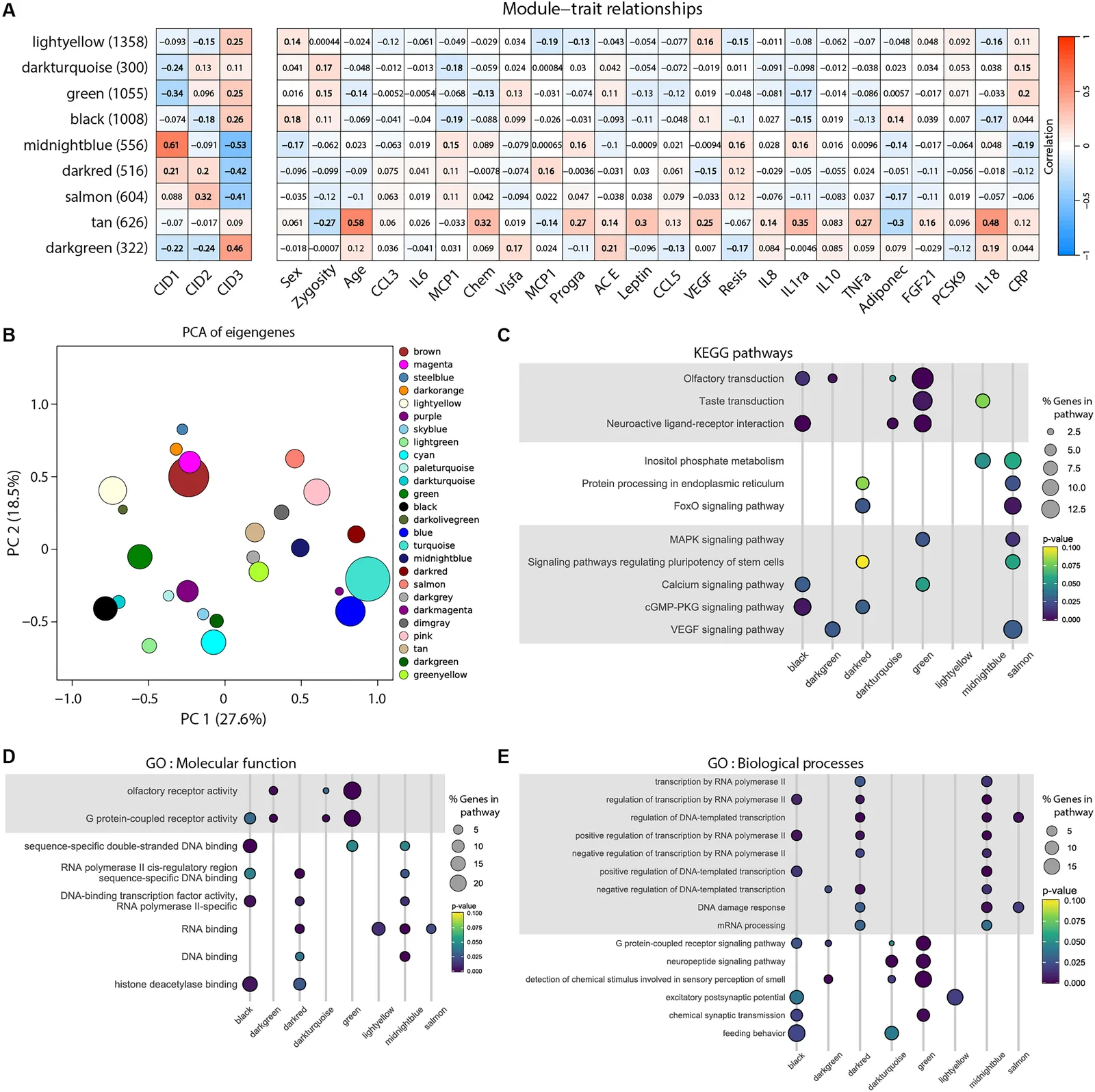
WGCNA of transcriptomic changes in adipose tissue. (**A**) Module-trait relationships for selected identified modules. Module-trait relationships were evaluated using correlations between module eigengenes and clinical traits. Module names follow the standard WGCNA color convention (*66*, *73*). Modules and their respective number of genes are displayed in rows, with columns containing CIDs, selected cytokines, and other traits. Cells show correlation values between a module and a trait, with the intensity and direction of the correlation mapped on a normalized color scale ranging from blue (negative) to red (positive). Significant correlations with *P* value ≤0.05 are highlighted in bold. The complete heatmap with all identified modules is found in fig. S3. (**B**) Principal components analysis of eigengenes for each module. Principal component 1 accounts for 27.6% of the total variance in the dataset, while principal component 2 explains 18.5% of the total variance. Circle sizes are scaled according to the number of genes present in a given module. (**C** to **E**) Enrichment analysis for gene ontology (GO): Biological Processes, GO: Molecular Function, and KEGG pathways for modules that correlate significantly to the CIDs [see (A)]. The size of the dots is scaled according to the % of genes of a given pathway that are present in the respective module with the *P* value of the enrichment mapped to a color scale.

To further investigate the impact of a WD on the genetic changes in adipose tissue, we performed a weighted gene coexpression network analysis (WGCNA), where genes were grouped into modules based on coexpression pattern similarity. Coexpression modules were labeled using the standard WGCNA color convention for visualization. These labels are identifiers rather than biological annotations, except that gray denotes genes not assigned to any module (*20*). We identified 26 modules named by colors, where multiple modules were highly correlated to dietary interventions, anthropometrics or inflammatory markers (fig. S3). The WGCNA algorithm automatically assigns a unique color label to each coexpression module. These color labels are arbitrary identifiers and used solely for identification purposes and do not convey any information about the biological function or content of the module. Using principal components analysis (PCA) of module eigengenes, a clear separation of the modules was apparent (Fig. 2B).

Figure 2A and fig. S3 display module-trait relationships for multiple clinical parameters as well as CIDs for each identified module. Eight modules were shown to significantly correlate and react to the dietary intervention. The modules midnight blue and dark red had a positive correlation to CID1 and a negative correlation to CID3, while dark turquoise, green, and dark green exhibited an inverse correlation. The modules light yellow, black, and salmon appeared to be heterogenous with varying significant correlations to CID2 and CID3 but no significant correlation to CID1. These modules thus include genes responding to the WD. Several other modules showed isolated significant correlations to different inflammatory clinical parameters with module tan displaying the most significant correlations to different cytokines and adipokines. However, it did not significantly correlate to CID1–3 and consequently its content was not attributable to the WD. This demonstrates a multitude of genetic changes occurring in adipose tissue, although no strong correlation could be shown for an inflammatory module reacting to the WD. We then looked further into the functional associations of the modules.

### Involvement of ORs

Module-based pathway analysis revealed a number of modules related to specific biological functions. The analysis highlighted modules in black, dark turquoise, dark red, salmon, and midnight blue related to regulation of transcription and metabolism as well as cell signaling and organization but also, unexpectedly, the involvement of olfactory transduction, OR activity and sensory perception, which was mostly found in the modules with negative correlation to CID1 and positive correlation to CID3: dark turquoise, green, and dark green. The modules black, light yellow, and dark red also, to a lesser extent, displayed associations to olfactory functions (Fig. 2, C to E, and tables S17 to S19).

**Fig. 3.**
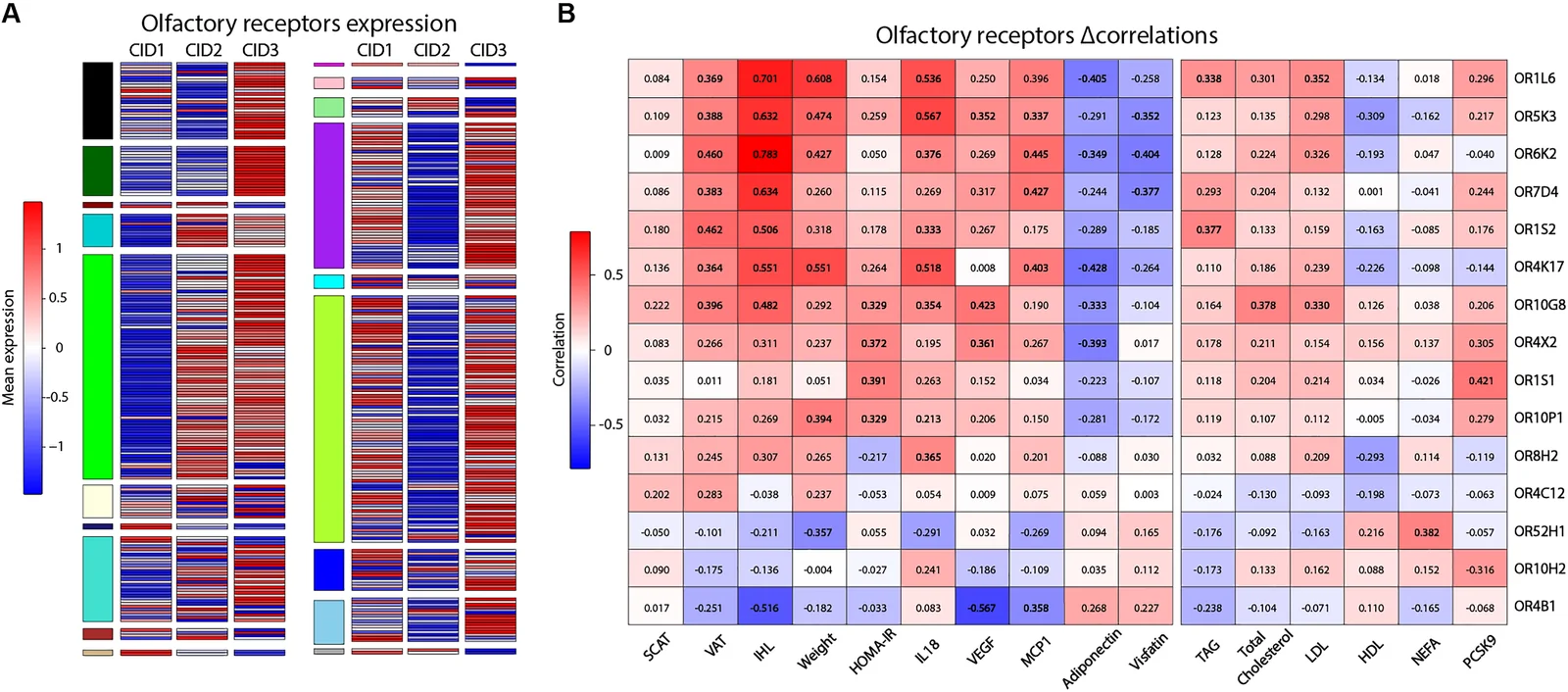
Overview of OR genes’ involvement in response to WD. (**A**) Heatmap of gene expression levels of ORs by CID. Genes are organized according to the modules they belong to. Gene expression levels are displayed as mean values per OR gene for CID1–3. (**B**) Heatmap demonstrating correlation changes of the top 15 differentially expressed OR genes and selected clinical parameters between CID1 and CID3. Correlation changes (Δ) with significant *P* values ≤0.05 are highlighted in bold. Additional clinical parameters are shown in fig. S4.

A closer look at the transcriptional changes of ORs revealed a, mostly continuous, up-regulation of many OR genes that were part of WGCNA modules with olfaction-related enrichment. Although, overall, most OR genes were up-regulated in response to the WD, many OR genes that were part of WGCNA modules without olfaction-related enrichment did not share the same profile of continuous change but were transiently down-regulated in CID2 and up-regulated in CID3 (Fig. 3A).

One-hundred fifty seven of 442 OR genes were significantly differentially expressed in one of the comparisons. One-hundred twenty six protein coding genes were retained, excluding 31 pseudogenes (table S20).

We then selected only protein-coding OR genes with absolute log_2_FC in CID1 versus CID3 of >0.5, leaving 15 genes. Most of them were up-regulated and showed continuous change.

An interaction network, created using the STRING database, showed only few known associations between these genes (*21*, *22*). OR7D4 was associated with OR10H2 and OR4C12, while OR1S1 and OR1S2 were associated with each other with a combined interaction score of >0.4, which is defined as “medium confidence.” No additional associations, passing the filter, could be shown for the other top OR genes (fig. S5).

Investigation of correlation changes of OR gene expression to cytokines and other clinical parameters in response to the dietary intervention then revealed noteworthy associations as displayed in Fig. 3B. Most of the top 15 significantly differentially expressed OR genes, after WD, showed a stronger correlation to several clinical parameters that are associated with obesity and inflammation and are generally associated with the metabolic syndrome, such as liver fat, visceral fat, HOMA-IR, IL-18, VEGF, MCP1, and LDL cholesterol. On the other hand, these genes decreased in correlation to the anti-inflammatory cytokine IL-10, the adipokines adiponectin, and eNAMPT and also HDL cholesterol. However, the OR genes OR4B1, OR10H2, and OR52H1 were clear exceptions to this pattern, reacting inversely regarding these correlations, both for positive and negative changes.

## DISCUSSION

The response to WD in healthy lean and mostly young people (mean age ± SD: 31 ± 14 years) showed moderate metabolic responses as reflected by the expected increase in total, LDL and HDL cholesterol already after 1 week, and a moderate decrease of insulin sensitivity. There was a significant average increase in body weight by 0.25 kg from CID1 to CID3, which we would not consider clinically relevant although we cannot exclude it. The significance of this small change resulted from the unidirectional parallel increase in most participants, probably reflecting the attractiveness of the WD. We previously reported small but significant increases in fasting glucose, fasting insulin, HOMA-IR, and AIRg, which were more pronounced in carriers of the angiotensin-converting enzyme (ACE) I/D polymorphism in response to the WD (*13*). The expected inflammatory response of plasma cytokines and chemokines was mostly transient with an increase of IL-6, IL-18, TNFα, and CCL5 after 1 week but return to baseline after 6 weeks and a small increase in the global inflammatory marker CRP. The assessment of adipose tissue immune cell composition by CIBERSORT did not indicate an increase in activated macrophages, but revealed moderate changes of CD4-T cells and a slight increase in NK cells. This indicates that healthy and lean subjects can compensate for the inflammatory stimulus provided by WD and aligns with previous studies in healthy normal-weight or overweight humans who ate hypercaloric high-fat diets without significant increases in insulin resistance or inflammatory markers (*9*, *11*, *23*). This raises the question why WD is closely linked to the metabolic syndrome and points to the role of obesity and ectopic fat (*24*).

In this work, we focused on the transcriptomic response of subcutaneous periumbilical adipose tissue, which surprisingly indicated an extensive remodeling after 1 and 6 weeks of WD, even in the absence of weight gain. This included genes involved in transcription, signal transduction, cellular and cytoskeletal organization, lipid metabolism, endocrine function, inflammation, sensory function, and perception. The abdominal subcutaneous adipose tissue gene expression thus reflects a pronounced response that contributes to the interorgan adaptations.

The pathway analysis of coregulated genes then uncovered a notable association between changes in G protein–coupled receptor activity, primarily ORs, and the consumption of the WD. A large group of OR genes was continuously up-regulated by the dietary intake. Importantly, these increases correlated with central markers of the metabolic syndrome such as hepatic fat accumulation, insulin resistance, and specific markers of inflammation such as IL-18, VEGF, and MCP1. Notably, the individual increases in body weight also correlated with the changes in ORs. The changes in ORs may play a hitherto unknown role in the early unfolding of these phenotypes upon high-fat diet before obesity develops. In agreement with these suggestions, an inverse association was found with the protective adipokine adiponectin and the cellular energy metabolism regulator eNAMPT (*25*–*27*).

ORs comprise the largest group of G protein–coupled sevenfold transmembrane receptors and were first identified in the olfactory sensory neurons of the olfactory epithelium and play an important role in human nutrition and well-being (*28*, *29*). They have since been located in numerous tissues involved in cardio-metabolic and renal diseases as well as the brain and were named ectopic ORs (*30*, *31*). They are mostly coupled to the second messenger adenylate cyclase but also to inositol triphosphate–dependent signal transduction in the periphery and adipose tissue (*32*). They are closely linked to the discrimination of thousands of tastants and odorants mediated by about 400 functional ORs in humans. Many ORs are activated by food derived metabolites and digestion products and thus are likely to mediate some of the beneficial or disadvantageous effects of food components (*30*). Numerous mouse and rat studies have established their pivotal role in cardiometabolic disease in animal models (*30*, *31*). The transfer to humans is limited by the moderate homology of rodent and human ORs. Responses to high-fat diet without weight gain in caused increased inflammation, insulin resistance, and loss of olfactory sensory neurons, suggesting that the high fat content of diet rather than obesity regulates the olfactory system (*33*). In mice, loss of sensory neurons prevented high-fat diet–induced obesity and improved insulin resistance and body fat distribution by increasing sympathetic activity while reducing food intake. Super-smeller mice due to whole body expression of the voltage-gated K(+) channel (Kv) subtype Kv1.3, showed resistance to diet-induced obesity while the deletion of insulin-like growth factor 1 (IGF-1) receptors in the olfactory system increased olfactory perception but caused insulin resistance and increased food intake (*34*). These studies underline the importance of the olfactory system in metabolic regulations but also reveal its complexity. Ectopic ORs may represent a separate system and elicit independent effects in peripheral tissues.

In humans, genetic variants of some ORs were associated with cardiometabolic disease, in particular type 2 diabetes mellitus (T2DM), obesity, and cardiovascular disease (*30*, *35*). However, the role of most ORs remains unclear since less than half have been deorphanized and their peripheral signaling pathways have not been studied in greater detail (*36*).

The extensive regulation of OR expression by high-fat diet in humans has not been reported before and came as a surprise. The characterization of the metabolic responses to the WD in the NUGAT study allows us to correlate the gene expression of the 15 most extensively up-regulated ORs to the metabolic responses. A strong and increasing correlation from CID1 to CID3 between ORs, liver fat content, and insulin resistance implies the involvement of many ORs in the early negative responses to WD. The correlation extended to IL-18, indicating a link to the TLR4-NLRP3-inflammasome pathway that was shown to respond to plasma lipids including cholesterol and ceramides (*2*). Both lipids increased continuously in the NUGAT study as shown by our lipidomics analysis (*14*). MCP1, a key chemokine attracting monocytes into adipose tissue was highly correlated with the most up-regulated ORs while other chemokines like CCL3/MIP1α and CCL5/RANTES showed an almost inverse correlation pattern (*37*, *38*). Some of the classical obesity-associated cytokines such as TNFα and IL-6 were only weakly correlated with the leading ORs in our setting. Notably, the adipokine adiponectin, which is established as a strong protective factor for cardiovascular and metabolic disease, correlated inversely with the same responses, underlining the consistency of these associations (*39*, *40*). The inverse association of adiponectin may be postulated to reflect some direct effects of adipocyte ORs on the expression of adiponectin, or it may represent a secondary response to the metabolic impairments.

The pattern of responses of adiponectin was shared by eNAMPT. Intracellular NAMPT regenerates intracellular nicotinamide adenine dinucleotide (NAD+), a central sensor and component of energy metabolism in mitochondria, the endoplasmatic reticulum, and the nucleus and is a cofactor of NAD-dependent metabolic and DNA-repair enzymes including sirtuin-histone deacetylases (*25*). eNAMPT has cytokine-like activity and modulates metabolic responses related to aging, metabolic dysfunction-associated steatotic liver disease, T2DM, and obesity by influencing lipid and glucose metabolism, redox stress, inflammation, and apoptosis (*41*, *42*). Current concepts place eNAMPT as the communicating link between hypothalamic energy control and the peripheral tissues including liver, muscle, and adipose tissue (*25*).

ORs are expressed in the hypothalamus, which integrates whole-body energy homeostasis and food intake (*34*, *43*). Odors sensed by olfactory neurons change the activation status of the hypothalamus and thereby affect appetite and satiety circuits that are directly linked to metabolic regulation and well-being (*28*). Moreover, by receiving olfactory inputs, the limbic region of the brain corrects emotional responses (*34*, *44*). Olfactory performance appears to diminish linearly with the BMI regarding odor thresholds, while odor discrimination and sensitivity were not altered according to a recent meta-analysis (*35*). Mutations in adenylate cyclase 3, which couples ORs, are associated with obesity in humans and gene variants of 23 human ORs were associated with obesity or fat distribution to date (*32*, *35*).

It is highly remarkable that eNAMPT shares the correlation profile of adiponectin with metabolic and inflammatory factors and is inversely correlated to increases of hepatic fat, IL-18 and MCP1. This aligns with the current view of eNAMPT as an important integrator of energy metabolism that interacts with ORs (*25*, *42*).

The increase in OR expression in healthy subjects seems to provide an important reason for the link of WD to obesity, particularly in view of the ability of healthy subjects to compensate for insulin resistance and inflammation in the absence of weight gain (*9*, *11*): The increased expression of ORs may enhance olfactory function and the attractiveness of high-fat energy-dense foods and therefore drive the intake of unhealthy diets, leading to obesity (*28*, *45*). ORs moreover appear to participate in metabolic and inflammatory regulation in peripheral tissues, which may be independent from effects in the nasal epithelium or central nervous system pathways.

Among the 15 most up-regulated ORs, single-nucleotide polymorphisms in *OR7D4*, a receptor for the porcine meat component androstenone and the pheromone androstadienone, have been linked to increased BMI, body fat percentage, waist circumference, susceptibility to hunger, and dietary restriction capacity, which aligns well with our associations (*35*, *46*). The other 14 top ORs in our study have not been deorphanized or characterized in further detail at present.

ORs are known to serve different functions, and 3 of the 15 most up-regulated ORs displayed an opposite association pattern to the metabolic and inflammatory biomarkers compared to the 12 others. They may be involved in health-promoting metabolic pathways. Our data provide a candidate list of human ORs associated with the metabolic and inflammatory functions of WD and offer potential targets for nutritional of pharmaceutical interventions. They also suggest that dietary effects on metabolic and inflammatory parameters may be mediated by ORs independent from the metabolic actions of macro- and micronutrient components themselves. This may particularly apply to red meat and also plant derived metabolites since their health-related effects are not fully explained at the mechanistic level and the over 400 ORs in humans are likely to respond to specific food components (*35*).

Ultimately, ORs are highly polymorphic leading to individual-specific responses as shown for *OR7D4* (*46*). A deeper understanding therefore paves the way for individual approaches to personalized nutrition, both at the level of gustatory preferences as well as regarding metabolic risks.

An important limitation is the short time frame of 2 × 6 weeks, since the metabolic and transcriptomic responses may differ over longer periods. We included only the local Western Europeans, and the responses may differ in other ethnicities. We investigated mostly lean subjects, and the responses are likely to differ in people with obesity. This was intended as we attempted to study the isolated effect of WD in the absence of secondary diseases that are typically associated with WD. A strength of the study is the relatively large group and the highly controlled intervention that first normalized the nutrition to a healthy diet to exclude differences due to dietary habits at baseline. The CIDs after 1 week and 6 weeks allowed us to discriminate acute versus longer term responses that indeed reflect a compensation for a specific set of parameters.

In conclusion, our study characterizes weight-independent inflammatory, metabolic, and transcriptomic responses to WD and reveals an extensive transcriptomic rearrangement in adipose tissue that involves a pronounced response of OR genes. Their highly consistent positive and negative association with metabolic responses, inflammatory, and protective adipokines implies ectopic ORs as contributors to the deleterious metabolic consequences of WD.

## MATERIALS AND METHODS

### Participants

The NUGAT study enrolled 46 pairs of twins, 34 monozygotic and 12 dizygotic and 58 female and 34 males (fig. S1). Zygosity was confirmed by DNA testing. Inclusion criteria were a BMI between 18 and 30 kg/m^2^, <3 kg/m^2^ BMI difference between the twins, age between 18 and 70 years and no documented pregnancy or severe disease.

The NUGAT study (ClinicalTrials.gov; NCT01631123) has been described in detail in previous publications (*12*–*15*). The study protocol was approved by the local Ethical Committee of the Charité Universitätsmedizin Berlin, Germany (EA4/021/09), in accordance with the Declaration of Helsinki of 1975, as revised in 1983. All participants gave written informed consent before the study.

A physical examination, anthropometry, and medical history were obtained and 12-hour fasted blood was collected at 8:00 a.m. on study days. Physical activity level was assessed by questionnaire. The metabolic, transcriptomic and inflammatory responses to a nutritional isocaloric intervention consisting of a shift from a low-fat to a high-fat diet was investigated. A timeline of the study is shown in Fig. 1A. The participants started the study with 6 weeks of a carbohydrate-rich, low-fat diet [30E% total fat with 10% saturated fatty acid (SFA), 10% mono-unsaturated fatty acid (MUFA), 10% poly-unsaturated fatty acid (PUFA), 55E% carbohydrates, and 15E% protein] establishing similar baseline conditions. They then switched to 6 weeks of a low-carbohydrate, high-fat diet (45E% total fat, with 18% SFA, 17% MUFA, 10% PUFA, 40E% carbohydrates, and 15E% protein). Both diets were adjusted to the individual energy requirements of the participants to maintain stable body weight. Energy requirements were estimated by indirect calorimetry (Vmax Encore metabolic cart; CareFusion, Yorba Linda, California) which was compared with the mean daily food intake calculated from a 5-day dietary record. Extensive dietary counseling, including individualized meal plans, was provided throughout the study with at least seven individual dietary consultations. Details of the nutrients used in the dietary phases are provided in tables S2 and S3. The subjects completed six dietary records of 5 to 6 days at specified times during the study as published previously (*14*). In addition, approximately 70% of the food was provided for 7 days before the CIDs. The low-fat diet was rich in whole-grain bread, fruits, vegetables, and legumes and plant oils, while the WD was enriched in white bread, butter, French fries, processed meat, and convenience foods (e.g., hamburger, fried sausage, and pizza).

### Clinical measurements

An oral glucose tolerance test was performed after inclusion into the study and routine clinical parameters were determined. At each CID, anthropometry was performed as described (*13*) and blood samples were drawn from the forearm vein after an overnight fast, centrifuged at 1.800*g* for 10 min at 4°C and serum was stored at −80°C until analysis. Determination of routine serum parameters including CRP was performed using an automated analyzer (ABX Pentra 4000; ABX, Montpellier, France). LDL cholesterol concentrations were calculated using the Friedewald equation. Insulin and C-peptide were determined with enzyme-linked immunosorbent assays (ELISAs) from Mercodia (Uppsala, Sweden). Chemokines and cytokines were measured using R&D ELISAs except for chemerin (Biovendor, Brünn, Czechia) and eNAMP/visfatin (AdipoGen, Liestal, Germany). Magnetic resonance spectroscopy was performed on a Magnetom Avanto 1.5T whole-body scanner (Siemens Healthcare, Erlangen, Germany) for quantification of liver fat content [intrahepatic lipids (IHL)] at CID1, CID2, and CID3. ivGTTs were performed in the fasting state in the morning as described in a previous publication (*13*).

For the clinical measurements displayed in Table 1, extreme outliers were identified and excluded using the interquartile range (IQR). Specifically, data points falling more than three times the IQR above the third quartile or below the first quartile were excluded. Data were checked for normality using the Shapiro-Wilk test and for heteroscedasticity with the Breusch-Pagan test (*47*, *48*). To account for data violating the assumptions of normality or homogeneity of variance, we then fit a linear mixed model to the data, with CID, age, zygosity, and sex as the fixed effect predictors and Patient ID as a random effect to control for repeated measures of the same individual with the “lme4” R package and used heteroscedasticity-consistent variance estimators using the “clubSandwich” R package (*49*, *50*). The overall effect of the intervention was assessed through a type III analysis of variance (ANOVA) with a χ2 test and post-hoc pairwise comparisons between CIDs were conducted and adjusted for multiple testing using the Benjamini-Hochberg method with the “emmeans” R package (*51*, *52*).

### Adipose tissue biopsy and RNA analysis

A biopsy of subcutaneous adipose tissue was performed after local anesthesia lateral to the umbilicus by needle aspiration. About 500 mg of adipose tissue were homogenized (Speed Mill, Analytik Jena, Jena, Germany). Total RNA was extracted by using the RNeasy Lipid Tissue Mini Kit (Qiagen, Hilden, Germany) according to the manufacturer’s protocol. For RNA quality assessment the RNA 6000 NanoLabChips and Agilent 2100 Bioanalyzer (Agilent Technologies, Santa Clara, CA) were used. Samples were amplified using the Amino Allyl MessageAmp II aRNA Amplification Kit (Ambion by Life Technologies, Carlsbad, CA) and hybridized onto Agilent Whole Human Genome 8×60K Gene Expression Microarrays (Agilent Technologies, Santa Clara, CA). Data have been uploaded to NCBI GEO (accession no. GSE62199). Real-time quantitative polymerase chain reaction was performed for selected genes to confirm the array data as described (*13*).

### Microarray data analysis

Clinical measurement and microarray data processing and analysis were performed in R version 4.3.2 (*53*). Using the RDAVIDWebService package required version 4.0.5 for compatibility reasons. Agilent Whole Human Genome 8×60K Gene Expression Microarray data were preprocessed and analyzed using the “limma” R package (*54*).

Annotations, including “probe ID,” “ensembl gene ID,” “HGNC gene symbol,” and “gene description,” were extracted with the “biomaRt” R package (*55*).

Density plots and PCAs were used to identify and exclude 23 outlier signals (*56*). Background correction and quantile normalization of the microarray data were performed with limma (*57*, *58*). Control probes, probes without corresponding gene symbols, as well as genes that were not well above the background signal were excluded (*59*). This reduced the number of genes from 62,976 to 46,687. For duplicate probe IDs, the mean expression value was used, resulting in 21780 unique genes.

When identifying DEGs, a paired analysis approach was employed to account for individual subject variability, specifically regarding age and sex. With the limma R package, linear models were fit to the gene expression data and empirical Bayes moderation was applied (*60*, *61*). This produced DEGs with *P* values for comparisons between each investigation day as well as an *F* statistic, combining the *t* statistics of every comparison (*54*). *P* values were adjusted for multiple testing using the Benjamini-Hochberg (false discovery rate) method (*52*).

To estimate the relative proportions of immune cell types from the adipose tissue gene expression profiles, the CIBERSORT deconvolution method was used, using the “IOBR” R package (*19*, *62*). CIBERSORT was performed with the default signature matrix LM22 (*19*). Only samples with a CIBERSORT *P* value of <0.05 were retained. We then fit a linear mixed model to the data, with the same methodology as described for the clinical measurements.

Pathway analysis was used with DAVID bioinformatics resources and the RDAVIDWebService package (*63*–*65*). For each comparison, genes with an adjusted *P* value below 0.05 were considered significantly differentially expressed. To reduce the foreground gene input to a usable amount, a threshold was set at an absolute log_2_FC of 0.5 and 0.8, respectively. For background genes, the default gene list was used. Pathway analysis was performed for the annotation categories Kyoto Encyclopedia of Genes and Genomes (KEGG), Gene Ontology (GO): Biological Process, GO: Molecular Function, and GO: Cellular Component.

Before performing additional pathway analysis, the up- and down-regulated genes were split into separate gene lists and the top 300 genes in terms of absolute FC were included, respectively. This was done to keep the gene input consistent across comparisons and resulted in absolute log_2_FC thresholds between 0.33 and 0.69.

WGCNA was used using the WGCNA R package (*66*, *67*). To relate the modules to the dietary interventions that are categorical variables, we generated binary indicators for each sample and diet as well as sex and zygosity. Additional phenotypes such as age and other clinical measurements were treated as continuous variables. Clinical measurements include CCL3 (MIP1α), IL-6, MCP1 (CCL2), chemerin, visfatin, pancreatic polypeptide, progranulin, ACE, leptin, CCL5 (RANTES), VEGF, resistin, IL-8, IL-1RA, IL-10, TNFα, adiponectin, FGF21, PCSK9, IL-18, and CRP. Five samples were excluded from the analysis as they were identified as outliers through hierarchical clustering. The analysis was performed with the “signed hybrid” scheme. The minimum number of genes per module was set to 100 genes, and modules with similarity values of 80% or above were merged. In the adjacency function, the power of 6 was selected as the soft threshold for the scale-free topology index *R*^2^, which reached a value of 0.84 and the mean connectivity being 37. Pathway analysis was performed for each module separately, based on all genes in the respective modules with no additional filters applied.

Correlation values were calculated using Spearman’s rank correlation coefficient (*68*). Delta correlations and corresponding *P* values were obtained through Fisher’s *r*-to-*Z* transformation and Zou’s confidence interval in the “cocor” R package (*69*–*72*).
