# Supplementary material for: Isocaloric switch from low-fat to Western high-fat diet triggers transcriptomic reprogramming and ectopic olfactory receptor responses
Source: Sci Adv. 2026 Aug 19;12(34):eaef5698. doi: 10.1126/sciadv.aef5698 (PMC13488902; doi:10.1126/sciadv.aef5698)
Supplement: Supplementary file 1 [file sciadv.aef5698_sm.pdf]

Science Advances
AAAS

Supplementary Materials for

Isocaloric switch from low-fat to Western high-fat diet triggers transcriptomic reprogramming and ectopic olfactory receptor responses

Jonathan Bertram *et al.*

Corresponding author: Andreas FH Pfeiffer, andreas.pfeiffer@charite.de

Sci. Adv. **12**, eaef5698 (2026)
DOI: 10.1126/sciadv.aef5698

The PDF file includes:

Figs. S1 to S5
Tables S1 to S3
Legends for tables S4 to S20

Other Supplementary Material for this manuscript includes the following:

Tables S4 to S20

Supplementary Figures

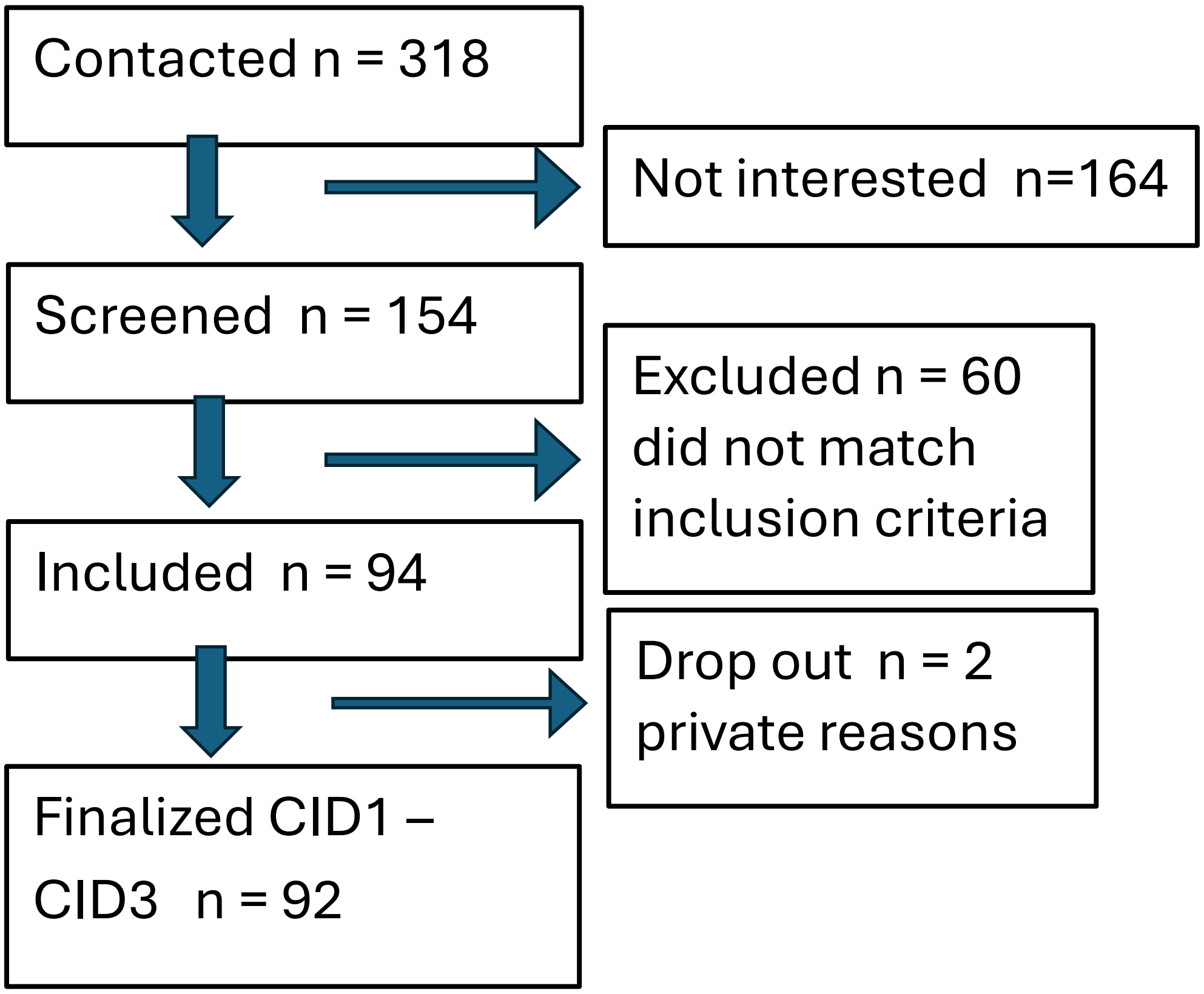

Figure S1. Schematic representation of the study recruitment. Respective numbers of participants from initial screening to completion of the dietary intervention.

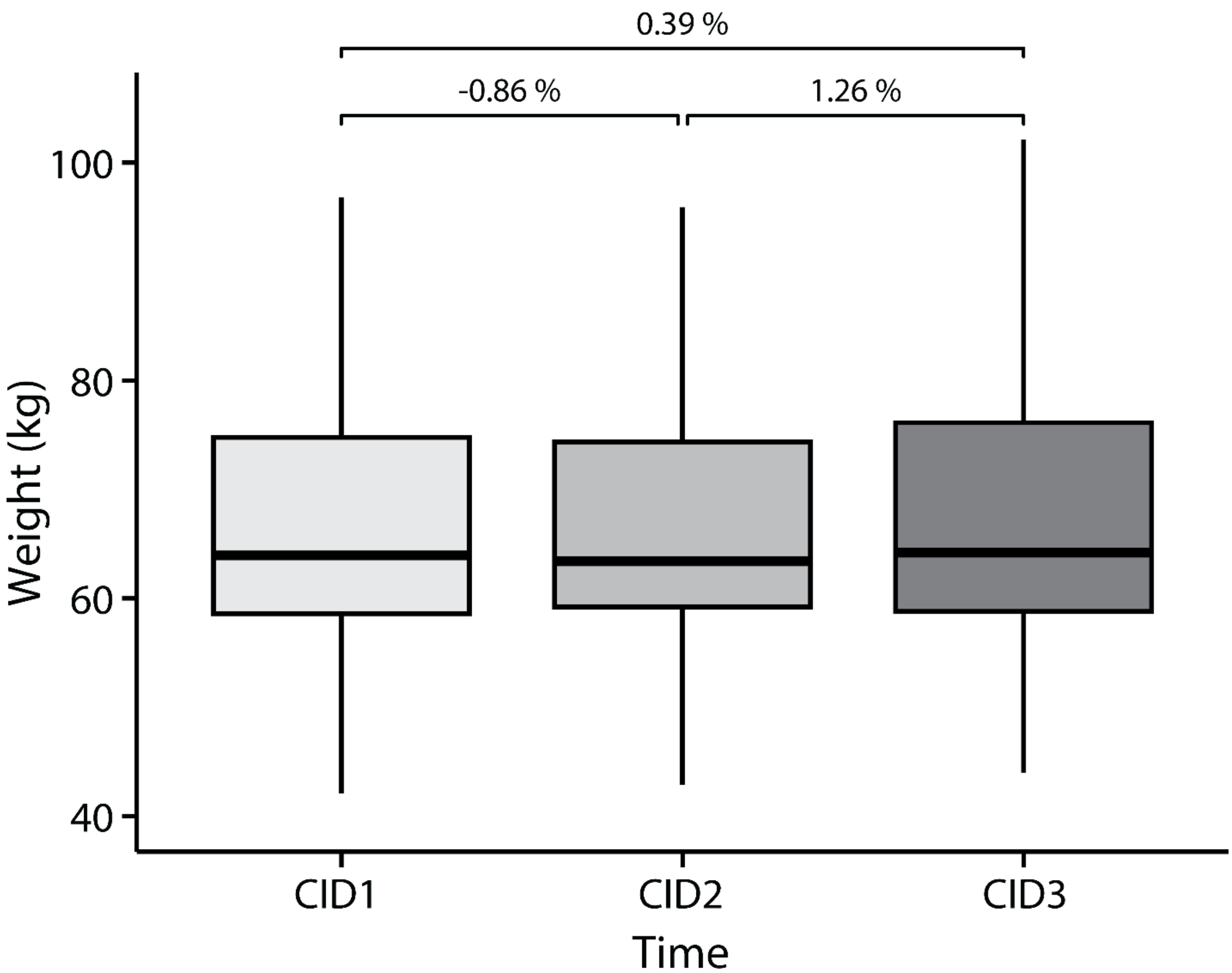

Figure S2. Weight distribution boxplots for all clinical investigation days. Relative changes were calculated using the median values.

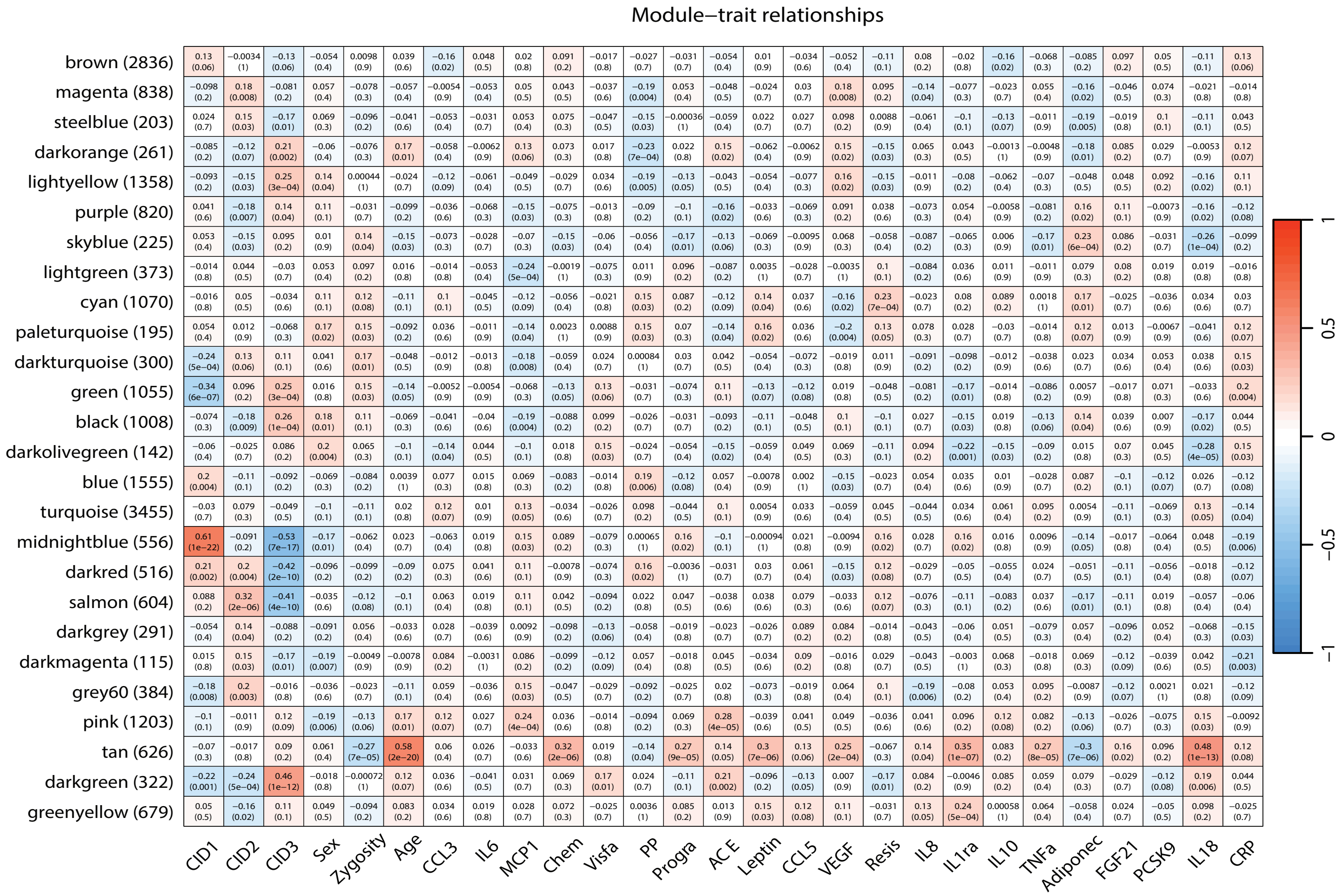

Figure S3. WGCNA module-trait relationship heatmap for all detected modules and phenotypes used. Every cell features the correlation value (top) and the corresponding p-value (bottom). The correlation values were colored according to a normalized color scale map.

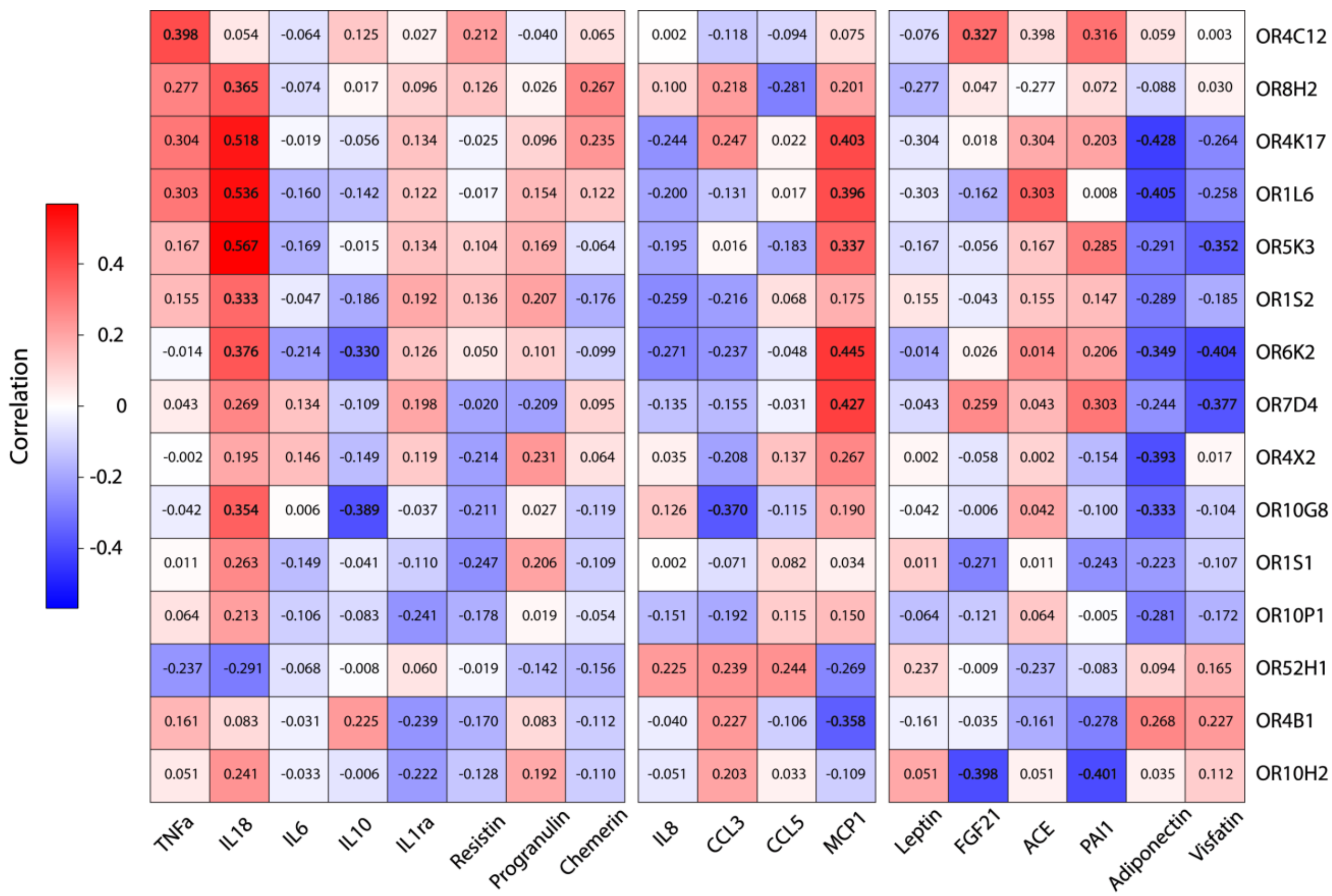

Figure S4. Correlation changes of OR genes and clinical parameters. Heatmap displaying the top 15 differentially expressed OR genes and their correlation with additional clinical parameters between CID 1 and CID 3. Correlation changes with significant p-values ≤ 0.05 are highlighted in bold.

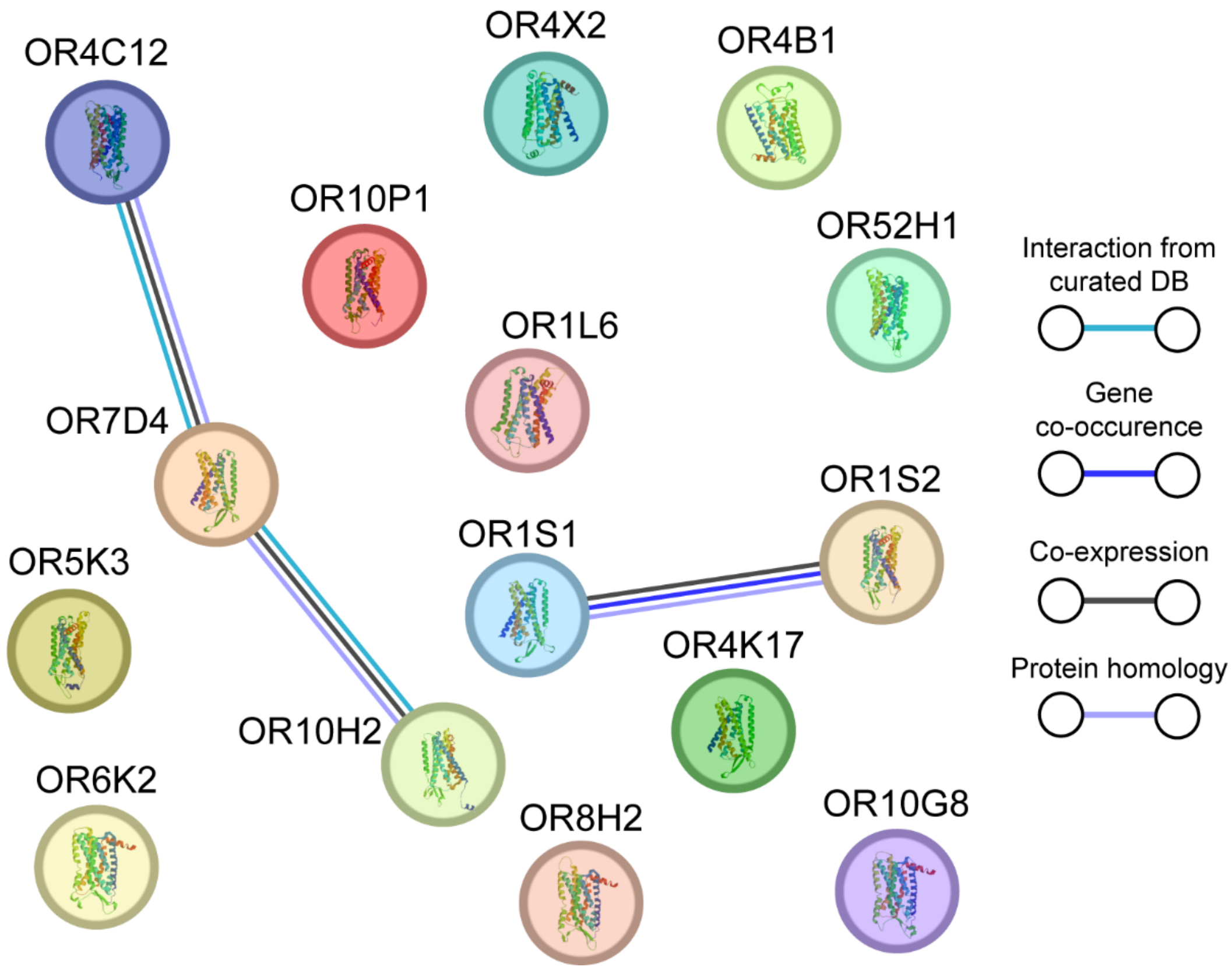

Figure S5. Protein-protein association network of the top 15 differentially expressed OR genes computed with StringDB (21, 22). Only interactions with a combined score of 0.4 or more are shown and are defined as "medium confidence".

Supplementary Tables

Cell type	Timepoint			p-value			
	CID1	CID2	CID3	CID 1v2	CID 2v3	CID 1v3	Overall
B cells naive	0.000 [0.000, 0.000]	0.000 [0.000, 0.000]	0.000 [0.000, 0.000]	0.333	0.333	0.333	0.384
B cells memory	0.090 [0.069, 0.119]	0.101 [0.070, 0.124]	0.098 [0.073, 0.139]	0.744	0.419	0.419	0.355
Plasma cells	0.015 [0.006, 0.024]	0.015 [0.009, 0.027]	0.012 [0.008, 0.022]	0.466	0.398	0.466	0.335
T cells CD8	0.030 [0.011, 0.061]	0.036 [0.007, 0.059]	0.041 [0.016, 0.068]	0.576	0.517	0.576	0.449
T cells CD4 naive	0.065 [0.034, 0.095]	0.076 [0.024, 0.117]	0.095 [0.056, 0.135]	0.431	0.194	**0.037**	0.048
T cells CD4 memory resting	0.146 [0.103, 0.190]	0.151 [0.109, 0.201]	0.100 [0.085, 0.130]	0.795	**0.008**	**0.002**	0.003
T cells CD4 memory activated	0.019 [0.000, 0.044]	0.017 [0.002, 0.045]	0.014 [0.002, 0.042]	0.944	0.901	0.901	0.795
T cells regulatory	0.000 [0.000, 0.000]	0.000 [0.000, 0.000]	0.000 [0.000, 0.000]	0.663	0.663	0.663	0.615
T cells gamma delta	0.132 [0.080, 0.193]	0.133 [0.096, 0.172]	0.129 [0.088, 0.169]	0.917	0.917	0.917	0.913
NK cells resting	0.000 [0.000, 0.000]	0.000 [0.000, 0.000]	0.000 [0.000, 0.000]	0.773	0.773	0.773	0.765
NK cells activated	0.000 [0.000, 0.012]	0.000 [0.000, 0.011]	0.009 [0.000, 0.018]	0.329	**0.032**	0.189	0.037
Monocytes	0.082 [0.043, 0.108]	0.061 [0.035, 0.104]	0.082 [0.054, 0.118]	0.375	0.182	0.286	0.116
Macrophages M0	0.036 [0.013, 0.082]	0.039 [0.010, 0.069]	0.038 [0.013, 0.061]	0.951	0.951	0.951	0.9
Macrophages M1	0.006 [0.000, 0.012]	0.008 [0.001, 0.018]	0.006 [0.002, 0.015]	0.24	0.979	0.273	0.217
Macrophages M2	0.029 [0.004, 0.057]	0.016 [0.000, 0.054]	0.026 [0.006, 0.051]	0.837	0.837	0.837	0.882
Dendritic cells resting	0.000 [0.000, 0.004]	0.000 [0.000, 0.003]	0.000 [0.000, 0.004]	0.541	0.926	0.541	0.513
Dendritic cells activated	0.000 [0.000, 0.000]	0.000 [0.000, 0.000]	0.000 [0.000, 0.000]	0.594	0.981	0.594	0.62
Mast cells resting	0.047 [0.031, 0.061]	0.045 [0.030, 0.078]	0.050 [0.036, 0.079]	0.347	0.962	0.347	0.359
Mast cells activated	0.000 [0.000, 0.000]	0.000 [0.000, 0.000]	0.000 [0.000, 0.000]	0.487	0.496	0.487	0.46
Eosinophils	0.000 [0.000, 0.000]	0.000 [0.000, 0.000]	0.000 [0.000, 0.000]	0.478	0.478	0.811	0.3
Neutrophils	0.199 [0.132, 0.257]	0.200 [0.137, 0.265]	0.205 [0.125, 0.245]	0.663	0.663	0.663	0.618

Table S1. Estimated relative proportions of cell type abundance in adipose subcutaneous tissue for each clinical investigation day using CIBERSORT (19). Proportions were normalized to a total of 1 and displayed as median [first quartile, third quartile] for each timepoint. Overall p-values were assessed through a Type III ANOVA with a χ^2 test and p-values for pairwise comparisons were obtained by comparing the estimated marginal means from the linear model and adjusted for multiple testing using the Benjamini-Hochberg method (52). The p-values for pairwise comparisons are robust regarding heteroscedasticity and significant values are highlighted in bold.

	High-Carbohydrate	High-Fat
**Macronutrient content (% of energy)**		
**Carbohydrates**	55	40
**Protein**	15	15
**Fat**	30	45
**Emphasized foods**	Wholemeal bread, wholemeal cereal, wholemeal pasta, brown rice, potatoes (unpeeled, boiled), legumes (beans, peas, lentils), fruits and vegetables (at least 5 times a day)	Wheat and rye bread, pasta, white rice, potatoes (fried potatoes, mashed potatoes, French fries), convenience foods (e. g. hamburger, fried sausage, pizza)
**Moderate intake**	Low-fat dairy products (skimmed milk, cheese, yoghurt) low-fat sausages (e.g. ham, chicken breast), low-fat meat and fish, eggs (boiled), vegetable oil	Fruits and vegetables, legumes, butter, high-fat dairy products (e. g. whole milk, full-fat yoghurt, cheese, cream), high-fat meat and sausages (e. g. salami, pork sausage, roast pork), eggs (scrambled egg, fried egg), sweets (e. g. chocolate, ice cream)
**Restricted**	High-fat meat and sausages (e.g., salami, pork sausage, roast pork), spread (butter, margarine), sweets, snacks, convenience foods	Low-fat dairy products (skimmed milk, cheese, yoghurt) low-fat sausages (e.g. ham, chicken breast), low-fat meat and fish
**Beverages**	Water, tea, coffee (unsweetened)	Water, tea, coffee (unsweetened)

Table S2. Dietary composition of study phases. Overview of food items and ingredients used in the dietary phases to ensure caloric and macronutrient consistency.

	High-Carbohydrate	High-Fat
**Breakfast**	Plenty of wholemeal bread + a little low-fat cheese or low-fat sausage or jam<br><br>Or<br><br>Wholemeal cereal + skimmed milk	A little wheat and rye bread + plenty of butter and high-fat cheese or high-fat sausage or chocolate spread
**Lunch**	Wholemeal pasta + braised vegetables + low-fat sauce	Mashed potatoes + fried sausage + buttered vegetables
**Dinner**	Wholemeal bread + low-fat cheese or low-fat sausage + salad	A little wheat and rye bread + plenty of butter and high-fat cheese or high-fat sausage + salad with feta cheese
**Snacks**	Fresh fruits and vegetables, low-fat yoghurt	Small sausages, full-fat yoghurt, pudding, chocolate

Table S3. Specific examples of employed dietary plans. Summary of typical meal compositions provided to participants during the respective dietary phases.

Supplementary Data Files

Supplementary Tables S4-S20 were too extensive to be included directly in this manuscript and are provided separately as multi-sheet Microsoft Excel files.

**Table S4**	Additional information for highlighted genes in the volcano plots from Fig. 1 D-F
**Tables S5-S10**	Detailed pathway analysis results for different time point comparisons with log_2FC cutoffs of 0.5 or 0.8 respectively
**Tables S11-S16**	Detailed pathway analysis results for different time point comparisons of the top 300 DEGs per direction of regulation
**Tables S17-S19**	Detailed GO and KEGG enrichment analysis results for WGCNA modules, corresponding to Fig. 2 C-E
**Table S20**	Full list of significantly differentially expressed, protein-coding olfactory receptor genes
